# Hesperetin modulates osteoprogenitor cells and macrophages under zoledronic acid and inflammatory stress

**DOI:** 10.1016/j.archoralbio.2026.106538

**Published:** 2026-02-05

**Authors:** Igor Paulino Mendes Soares, Owen Liepman, Caroline Anselmi, Sarah Chang, Josimeri Hebling, Renan Dal-Fabbro, Marco C. Bottino

**Affiliations:** a Department of Cariology, Restorative Sciences, and Endodontics, School of Dentistry, University of Michigan, Ann Arbor, MI, USA; b Department of Dental Materials and Prosthodontics, São Paulo State University (UNESP), School of Dentistry, Araraquara, Brazil; c Department of Morphology and Pediatric Dentistry, São Paulo State University (UNESP), School of Dentistry, Araraquara, Brazil; d Department of Biomedical Engineering, College of Engineering, University of Michigan, Ann Arbor, MI, USA

**Keywords:** Zoledronic acid, Flavonoids, Bone regeneration, Immunomodulation, Coculture techniques

## Abstract

**Objectives:**

To investigate the osteogenic and immunomodulatory effects of hesperetin (HT) on alveolar bone-derived mesenchymal stem cells (aBMSCs) and macrophages under zoledronic acid (ZA) and inflammatory stress.

**Design:**

aBMSCs were exposed to ZA (0–10 μM) for 3 days, followed by HT (0–1000 μM) for 3 days. Cell viability was assessed for 3 days of treatments, and osteogenic activity was evaluated by alizarin red quantification at 14 and 21 days. THP-1-derived macrophages were polarized to M1 using lipopolysaccharides (LPS, 1 μg/mL) and treated with HT (1–50 μM) to evaluate cell viability and synthesis of cytokines (ELISA). A co-culture system of aBMSCs and macrophages (1:1 ratio) was established under inflammatory stimulation (LPS ± 20 μM HT) to assess cell viability, cytokine release and mineralized matrix formation. Data were analyzed by ANOVA/post-hoc tests (α = 5 %).

**Results:**

ZA significantly reduced aBMSCs viability and mineralization in a dose-dependent manner. HT (5–50 μM) enhanced mineralization in healthy aBMSCs and partially restored it after ZA exposure. In M1 macrophages, HT (5–20 μM) decreased TNF-α, IL-1α, and IL-6 synthesis without affecting viability. In inflammatory co-cultures, HT (20 μM) preserved cell viability, increased mineralized matrix deposition, and reduced cytokine release compared to LPS-only controls.

**Conclusions:**

This study evidenced that HT can concurrently stimulate osteogenic differentiation and suppress inflammatory responses under ZA- and LPS-induced stress. HT emerges as a promising osteoimmunomodulatory adjuvant to enhance bone regeneration and mitigate bisphosphonate-related osteonecrosis.

## 1. Introduction

Bone regeneration depends on a balance between osteoclast-mediated resorption and osteoblast-driven bone formation (Salhotra et al., 2020). Disruption of this balance by aging, inflammation, or pharmacological interventions compromises regenerative capacity and increases the risk of graft or implant failure (H. Liu et al., 2025; Sulaiman et al., 2023). Zoledronic acid (ZA), a nitrogen-containing bisphosphonate widely prescribed for osteoporosis, metastatic bone disease, hypercalcemia, and Paget’s disease, is highly effective at suppressing osteoclast activity and increasing bone density (Ebetino et al., 2022; H. Liu et al., 2025). However, its strong affinity for hydroxyapatite and prolonged bone retention (~10 years) can impair osteoblast function and the osteogenic differentiation of progenitor cells, particularly in alveolar bone, where turnover is high (Basso et al., 2013; H. Liu et al., 2025; Patntirapong et al., 2012).

Medication-related osteonecrosis of the jaw (MRONJ) is a severe side effect of long-term bisphosphonate therapy, characterized by exposed, infected, and necrotic bone (Al-Omari et al., 2023). Its incidence ranges from 0.4 % to 21 %, depending on treatment regimen and patient risk factors such as dental procedures or oncologic comorbidities (Jelin-Uhlig et al., 2024). In ZA-compromised bone, local insults can facilitate bacterial invasion and intensify oxidative stress, driving a sustained M1-dominant inflammatory response that elevates cytokine release and osteoclastic activity while concurrently suppressing osteogenesis and vascularization, processes that collectively contribute to MRONJ development (Bagan et al., 2013, 2014; de Barros Silva et al., 2016, 2017; Paschalidi et al., 2021; Roato et al., 2023). These mechanisms underscore the importance of prevention over treatment and the need for strategies that modulate inflammation while enhancing osteogenic responses in patients receiving long-term bisphosphonate therapy (Jelin-Uhlig et al., 2024).

Flavonoids have emerged as promising therapeutic agents due to their potent osteogenic, antioxidant, and anti-inflammatory properties (Jomova et al., 2025; Shanmugavadivu et al., 2022). By scavenging reactive oxygen species and attenuating pro-inflammatory signaling, flavonoids can mitigate oxidative stress and chronic inflammation, which are two key factors that contribute to impaired bone healing (Jomova et al., 2025; Mendes Soares et al., 2024). Among them, hesperetin (HT), a citrus-derived flavanone, has been shown to enhance osteoblast differentiation, promote mineralization, and reduce inflammatory responses (Kim et al., 2013; L. Liu et al., 2021; Mancim-Imbriani et al., 2024; Mendes Soares et al., 2024; Salehi et al., 2022; Xie et al., 2023; Xue et al., 2017). This dual osteogenic-immunomodulatory profile positions HT as a candidate to offset ZA-related impairments in bone repair, yet its efficacy under inflammatory stress has not been established. Here, we test HT as an osteoimmunomodulator *in vitro*, assessing osteogenic rescue in ZA-exposed progenitors, suppression of M1 cytokines, and promotion of mineralized matrix under inflammatory conditions.

## 2. Materials and methods

### 2.1. Cell cultures

Alveolar bone mesenchymal stem cells (aBMSCs) were previously isolated from alveolar bone marrow explants from six patients (age range: 25–70 years) undergoing routine dental implant placement, following University of Michigan Institutional Review Board approval (IRB #HUM00064770), and according to previously reported protocols (Mason et al., 2014; Zou et al., 2022). All donors were systemically healthy and were not taking medications known to influence bone metabolism. The cells were cultured in complete nucleoside-free α-MEM (Gibco, Carlsbad, CA, USA) supplemented with 15 % fetal bovine serum (FBS; Gibco) and 1 % penicillin/streptomycin (Gibco) until they reached approximately 90 % confluence. Flow cytometry was performed to confirm CD73^+^ , CD90^+^, and CD105^+^ mesenchymal stem cell markers, and their multipotency was determined by Alcian Blue (chondrogenic), Oil Red O (adipogenic), and Von Kossa (osteogenic) staining (Mason et al., 2014; Zou et al., 2022). Passages from #5 to #8 were used for all the experiments using 0.25 % trypsin-EDTA (Gibco) for subculture procedures.

The THP-1 human monocytic cell line (TIB-202, LOT: 70061669, American Type Culture Collection, ATCC) was grown in RPMI 1640 Medium (ATCC modification, Gibco) supplemented with 0.05 mM 2-mercaptoethanol, 1 % penicillin/streptomycin (Gibco), and 10 % FBS (Gibco). Fresh medium was added every 2 days and completely renewed every 7 days, maintaining a density of around 1.5 × 10^6^ cells/mL.

### 2.2. Establishment of zoledronic acid pre-treatment of aBMSCs

Zoledronic acid (ZA) monohydrate (#SML0223, Sigma-Aldrich, St. Louis, MO, USA) was diluted in ultrapure water to make a 10 mM stock solution, which was stored at −80 °C and used within 6 months. The stock ZA solution was diluted to a 1 mM working solution that was further diluted in osteogenic medium composed of a complete medium supplemented with 10 mM β-glycerophosphate, 100 nM dexamethasone, and 50 μg/mL ascorbic acid to mimic the microenvironment required for differentiation and mineralization.

Cells were seeded (1 × 10^4^ cells/well) in 48-well plates and treated with ZA after 24 h. Based on previous *in vitro* findings (Patntirapong et al., 2012) and clinically relevant levels of ZA found in human saliva (Scheper et al., 2009), zoledronic acid pre-treatment of aBMSCs was initially tested using a challenging protocol with the concentrations of 0 – control, 1 μM, 5 μM, 10 μM, and 50 μM for 7 days. Cell viability was assessed 7 and 14 days after the first ZA exposure. Briefly, cells were incubated with 10 % alamarBlue reagent diluted in SBF-free α-MEM for 3 h at 37 °C and 5 % CO_2_ (Mendes Soares et al., 2024). After incubation, the fluorescence intensity of 100 μL of the media was determined at 555 nm excitation and 590 nm emission (SpectraMax iD3, Molecular Devices LLC, San Jose, CA, USA). The average fluorescence intensity of the control group was considered as 100 % individually for each evaluation period. Cells were washed with PBS and the osteogenic medium was replaced to maintain the culture for the other experiments. Fourteen and 21 days after cell seeding, the mineralized matrix deposition was assessed. Cells were fixed with 70 % ethanol, rinsed with deionized water, and stained with 40 mM alizarin red S solution (pH 4.2; Sigma-Aldrich) for 15 min with shaking (Mendes Soares et al., 2024). Background staining was removed by rinsing three times with deionized water. Calcium deposits were photographed and then solubilized in a cetylpyridinium chloride solution (10 mM; pH 7.0; Sigma-Aldrich) to measure absorbance at 570 nm (SpectraMax iD3). Cells cultured only with osteogenic media were used as a control, representing 100 % of mineralization.

The prolonged ZA exposure for 7 days led to excessive cytotoxicity, compromising cell viability and making it difficult to assess its effects on mineralization. To improve cytocompatibility while maintaining its inhibitory effect, we refined the protocol by reducing the ZA concentration range (0 – control, 0.5 μM, 1 μM, 2.5 μM, 5 μM, and 10 μM) and shortening the exposure time to 3 days, also based on previous findings (Patntirapong et al., 2012). This allowed a more controlled and physiologically relevant assessment of chronic administration of ZA on aBMSCs. Cells were exposed to ZA concentrations for 3 days, with the ZA treatments replaced every 24 h. Cell viability was assessed 1, 3, and 5 days after the first ZA exposition using alamarBlue, and the mineralized matrix deposition was evaluated 14 and 21 days after cell seeding using alizarin red, as previously detailed.

### 2.3. Modulatory effects of hesperetin on healthy and zoledronic acid-challenged aBMSCs

Hesperetin (HT, ≥95 % purity, W431300, Sigma-Aldrich) was dissolved at 10 mM in dimethyl sulfoxide (DMSO) (Sigma-Aldrich) to obtain a stock solution that was filter sterilized (0.22 μm pore size), stored at − 80 °C, and used within 6 months (Mendes Soares et al., 2024). A 1 mM working solution (in PBS) was prepared for the experiments. The effects of HT were investigated on healthy and ZA-challenged aBMSCs. Cells were seeded (1 ×10^4^) in 48-well plates and treated with ZA (5 μM) or basal medium (control) for 3 days. After that, cells were treated with 0 – control, 1 μM, 5 μM, 10 μM, 20 μM, 50 μM, 100 μM, 200 μM, 500 μM, and 1000 μM HT diluted in basal medium (α-MEM; Gibco). Cell viability was evaluated every 24 h using alamarBlue and the concentrations were renewed for up to 3 days (72 h). After HT stimuli were removed, cells were cultured in an osteogenic medium, renewed every other day. The mineralized matrix deposition was evaluated 21 days after cell seeding using alizarin red. Cells cultured only with osteogenic media were used as a control, representing 100 % of mineralization.

### 2.4. Establishment of an in vitro inflammatory microenvironment and modulation by hesperetin

The THP-1 human monocytic cell line was differentiated into macrophages (M0 phenotype) by incubation with 100 nM phorbol 12-myristate 13-acetate (PMA; Sigma-Aldrich) for 48 h in complete RPMI medium (Lund et al., 2016). Differentiation of THP-1 cells into macrophages was confirmed by the transition from a non-adherent suspension morphology to a firmly adherent macrophage-like phenotype following PMA treatment, as previously described (Lund et al., 2016). After differentiation, cells were washed twice with PBS and cultured for an additional 24 h in fresh medium without PMA to stabilize the M0 phenotype. To induce M1 polarization, cells were stimulated with lipopolysaccharides (LPS; *Escherichia coli* 0111:B4; Sigma-Aldrich) at 0.1 μg/mL and 1 μg/mL for 24 h.

For immunofluorescence analysis, cells were fixed with 4 % paraformaldehyde (Sigma-Aldrich) for 15 min, permeabilized with 0.1 % Triton X-100 for 5 min, and blocked with 1 % bovine serum albumin (BSA; Sigma-Aldrich) for 1 h at room temperature. Cells were then incubated overnight at 4 °C with anti-iNOS antibody (1:50; Abcam, Cambridge, MA, USA), followed by Alexa Fluor 488-conjugated secondary antibody (1:1000; Abcam). Nuclei were counterstained with 4’,6-diamidino-2-phenylindole (DAPI; Invitrogen), and actin filaments were stained using ActinRed 555 ReadyProbes reagent (Invitrogen) for 20 min, followed by PBS washing. Images were acquired using a fluorescence microscope (Echo Revolve, BICO Company, San Diego, CA, USA). To confirm the functional M1 phenotype, culture supernatants were collected after 24 h and analyzed for cytokine synthesis (TNF-α and IL-6) using specific human ELISA kits (Biolegend, San Diego, CA, USA), following the manufacturer’s instructions. Absorbance was measured at 450 nm (SpectraMax iD3). Cytokine concentrations were calculated using calibration curves.

To assess the modulatory effects of HT, M1-polarized macrophages were treated with HT at 1–50 μM for 24 h in the presence or absence of 1 μg/mL LPS, since this concentration elicited a marked M1 phenotype. Cell viability was determined using the alamarBlue assay (Invitrogen). After 24 h of treatment, culture supernatants were collected and analyzed for cytokine synthesis (TNF-α, IL-1α, and IL-6) using specific human ELISA kits (Biolegend, San Diego, CA, USA), as previously described.

### 2.5. Establishment of a co-culture system to mimic inflammatory and ZA-challenged bone microenvironments

To establish the co-culture system, THP-1 monocytes were differentiated into macrophages (M0 phenotype) by incubation with 100 nM PMA (Sigma-Aldrich) for 48 h, followed by 24 h in PMA-free medium. In parallel, aBMSCs were cultured in osteogenic medium (α-MEM supplemented with 10 mM β-glycerophosphate, 100 nM dexamethasone, and 50 μg/mL ascorbic acid) for 3 days to induce a pre-osteoblastic fate. After this period, aBMSCs were detached and seeded together with M0-differentiated THP-1 cells at a 1:1 ratio to establish the co-culture system. Three experimental conditions were initially tested:

1. **Basal model:** aBMSCs + THP-1 macrophages without additional stimuli.
2. **Inflammatory model:** co-cultures exposed to 1 μg/mL LPS for 24 h, to simulate a chronic inflammatory microenvironment.
3. **Inflammatory + ZA model:** co-cultures established with aBMSCs previously exposed to 5 μM zoledronic acid for 3 days before co-culture, followed by LPS challenge. This condition aimed to mimic clinical scenarios of patients under bisphosphonate therapy but resulted in excessive cytotoxicity, precluding further analyses.

Cell viability in co-culture was assessed using alamarBlue assay at 24 h and 72 h after treatments, as described in Section 2.2. Cell morphology and interactions within the co-culture were qualitatively evaluated by brightfield microscopy (ECHO, San Diego, CA, USA).

### 2.6. Modulatory effects of hesperetin on the inflammatory co-culture system

Given the high aggressiveness of the inflammatory + ZA condition, only the inflammatory model was selected for subsequent experiments with HT. Briefly, aBMSCs were pre-cultured for 3 days in osteogenic medium, and THP-1 monocytes were differentiated into macrophages (M0 phenotype) by PMA exposure (100 nM, 48 h) followed by 24 h in PMA-free medium. Both cell types were then seeded together at a 1:1 ratio to establish co-cultures.

Experimental groups were defined as follows: negative control (NC, co-culture without LPS stimulation); positive control (PC, co-culture stimulated with LPS at 1 μg/mL for 24 h); and LPS + hesperetin (20 μM HT added simultaneously with LPS for 24 h). After 24 h of treatment, the cells were washed with PBS, and the cultures were maintained in osteogenic medium, which was renewed every 2 days, for up to 21 days. Cell viability was assessed at 1, 3, and 7 days after, using the alamarBlue assay. In parallel, culture supernatants collected at 24 h were analyzed for cytokine secretion (TNF-α, IL-1α, and IL-6) using human ELISA kits (Biolegend), following the manufacturer’s instructions. Mineralized matrix deposition was quantified at 21 days by alizarin red staining.

### 2.7. Data analyses

Data were initially tested for normality (Shapiro-Wilk tests) and homoscedasticity (Brown-Forsythe tests) to guide the selection of appropriate statistical methods. Depending on the dataset, the following analyses were applied: two-way ANOVA/Sidak’s test, one-way ANOVA/Tukey’s test, and one-way ANOVA/ Dunnett’s test. Data were expressed as mean ± standard deviation (SD), and a significance level of 5 % was adopted for all analyses. Statistical analyses were performed using GraphPad Prism version 10.3.1 for Mac (GraphPad Software, San Diego, CA, USA).

## 3. Results

### 3.1. Establishment of zoledronic acid pre-treatment of aBMSCs

**Fig. 1.**
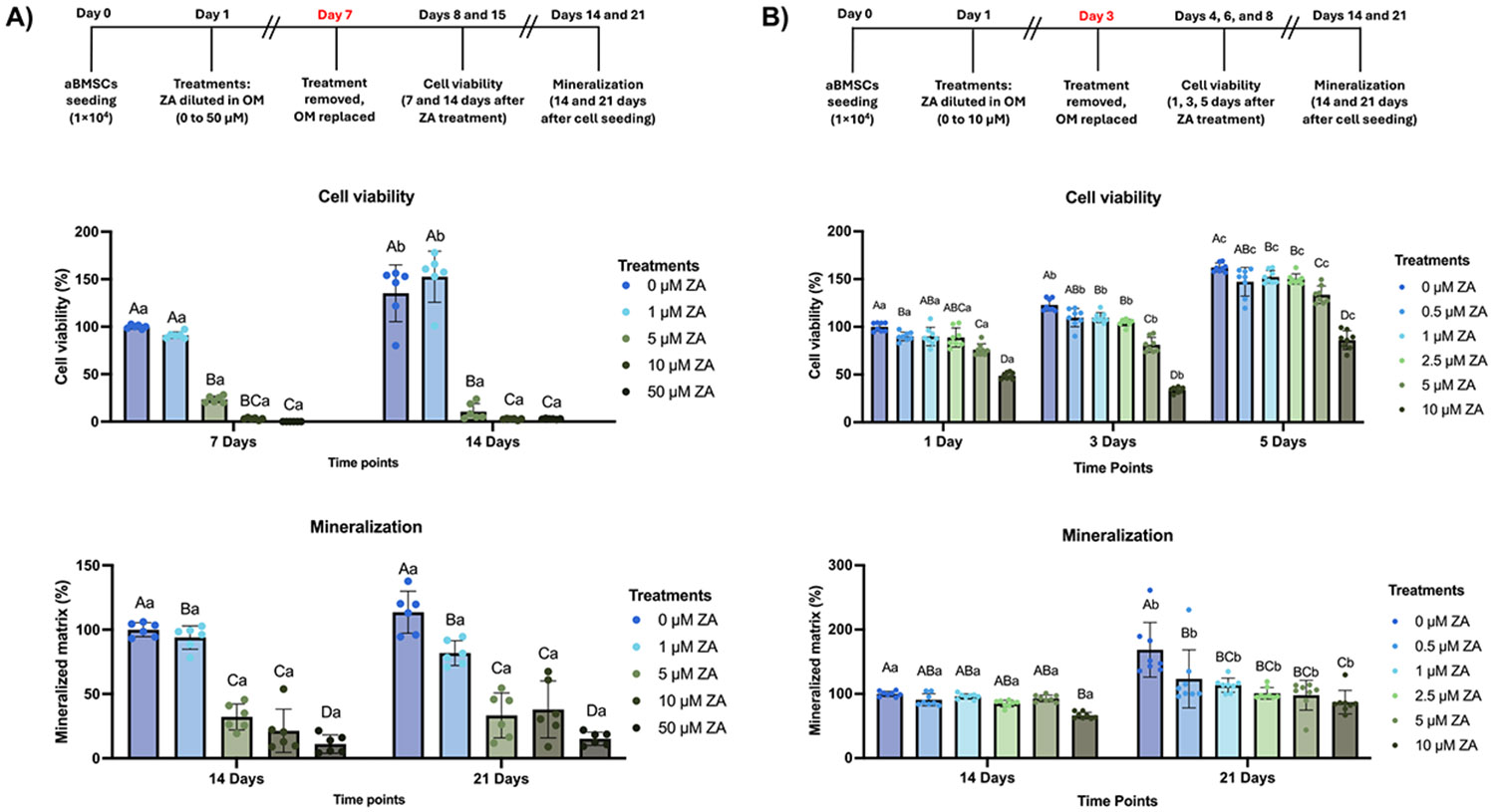
Experimental designs for the establishment of zoledronic acid (ZA) pre-treatment of alveolar bone-derived mesenchymal stem cells (aBMSCs). **(A)** Cells were seeded and treated with varying concentrations of ZA for 7 days. Then, the medium was replaced with osteogenic medium (OM) and refreshed every 2 days. Cell viability was assessed at days 7 and 14 after the first day of ZA exposure. Mineralization was evaluated 14 and 21 days after cell seeding. Data are presented as means ± SD (n = 6). **(B)** Cells were seeded and treated with varying concentrations of ZA for 3 days, after which the medium was replaced with OM and refreshed every 2 days. Cell viability was assessed 1, 3, and 5 days after ZA exposure. Mineralization was evaluated 14 and 21 days after cell seeding. Data are presented as means ± SD (n = 8). For all graphs, uppercase letters indicate statistical comparisons between treatments within the same time point, while lowercase letters compare treatments across different time points. Different letters denote statistically significant differences (Two-way ANOVA/Sidak’s test, α=5 %).

Extended exposure of aBMSCs to ZA (1–50 μM for 7 days) significantly reduced cell viability in a concentration- and time-dependent manner, with marked cytotoxic effects observed at both 7 and 14 days. Under these conditions, mineralized matrix formation was almost completely suppressed at 14 and 21 days (Fig. 1A). To improve cytocompatibility, the protocol was refined to shorter exposure (3 days) and lower concentrations (0.5–10 μM). This approach maintained higher viability while still showing a dose-dependent reduction in metabolic activity. Even under this refined protocol, ZA pre-treatment significantly impaired mineralized matrix deposition at 14 and 21 days compared with untreated controls (Fig. 1B). These results confirmed the inhibitory effects of ZA on aBMSC viability and osteogenesis while establishing experimental conditions suitable for subsequent modulation studies.

### 3.2. Modulatory effects of hesperetin on healthy and ZA-challenged aBMSCs

**Fig. 2.**
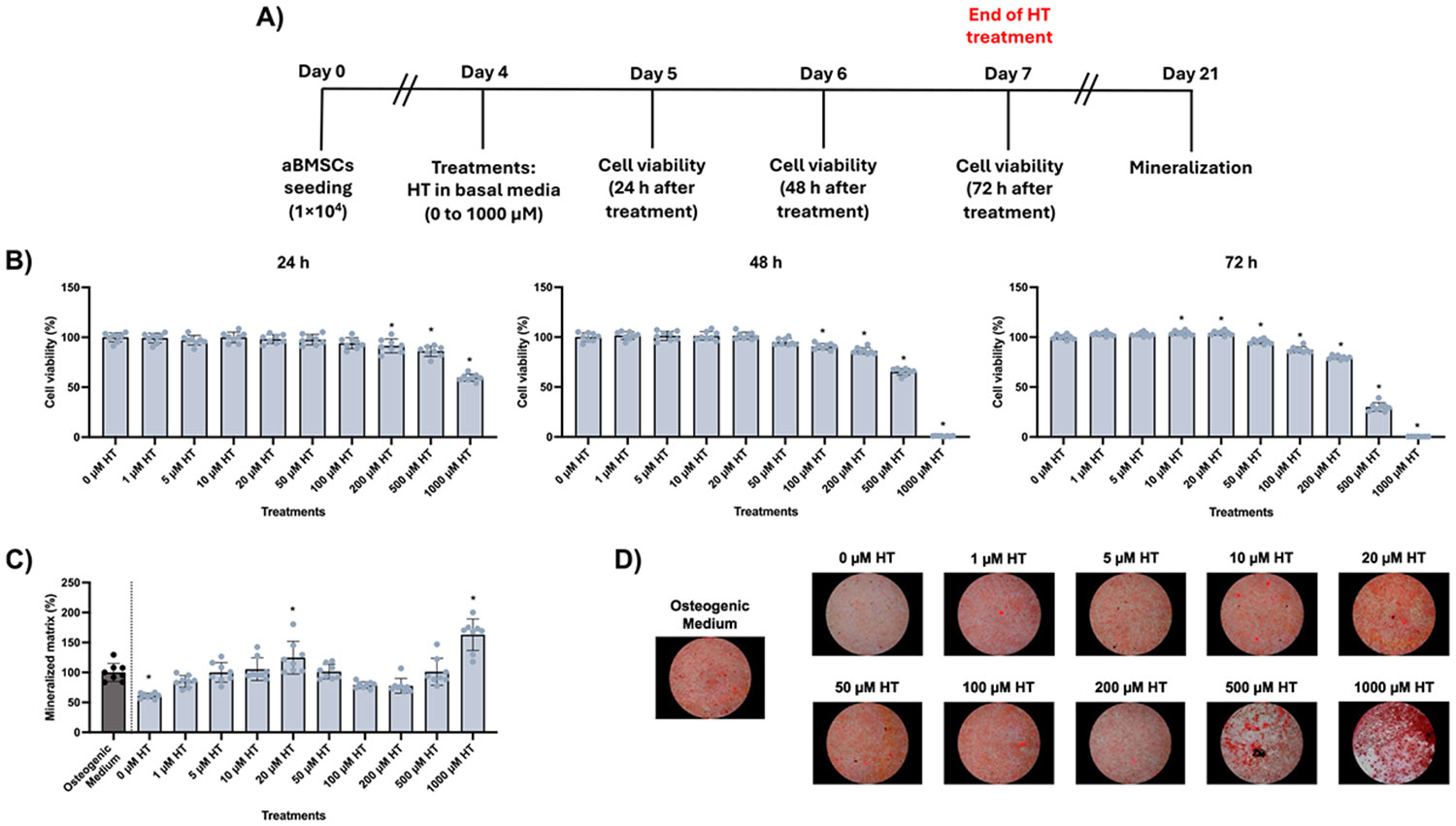
Modulatory effects of hesperetin (HT) on healthy alveolar bone-derived mesenchymal stem cells (aBMSCs). **(A)** Experimental design: Cells were seeded and cultured in basal medium for 3 days. Then, the medium was replaced with basal medium containing varying concentrations of HT for 72 h. Cell viability was assessed 24, 48, and 72 h after the HT exposure, which was renewed daily. After that, treatments were replaced with osteogenic medium (OM), which was renewed every other day, and mineralization was evaluated 21 days after cell seeding. **(B)** Cell viability and **(C)** mineralization results. Data are presented as means ± SD (n = 8) and calculated as a percentage of 0 μM HT for cell viability and osteogenic medium for mineralization, respectively, as controls. Asterisks indicate statistical difference compared to the respective controls (One-way ANOVA/Dunnett’s test, α=5 %). **(D)** Representative images of cells stained with Alizarin red solution, showing the red calcium deposits.

The effects of HT were first evaluated in healthy aBMSCs (Fig. 2A). HT exposure for 72 h was not cytotoxic at concentrations up to 200 μM (≥ 79.6% cell viability), while higher concentrations (≥ 500 μM) reduced metabolic activity by more than 70 % (Fig. 2B). Regarding osteogenic differentiation, concentrations between 5 and 50 μM increased mineralized matrix deposition compared to untreated controls. Representative images confirm the higher calcium deposition in these conditions (Fig. 2C-D).

**Fig. 3.**
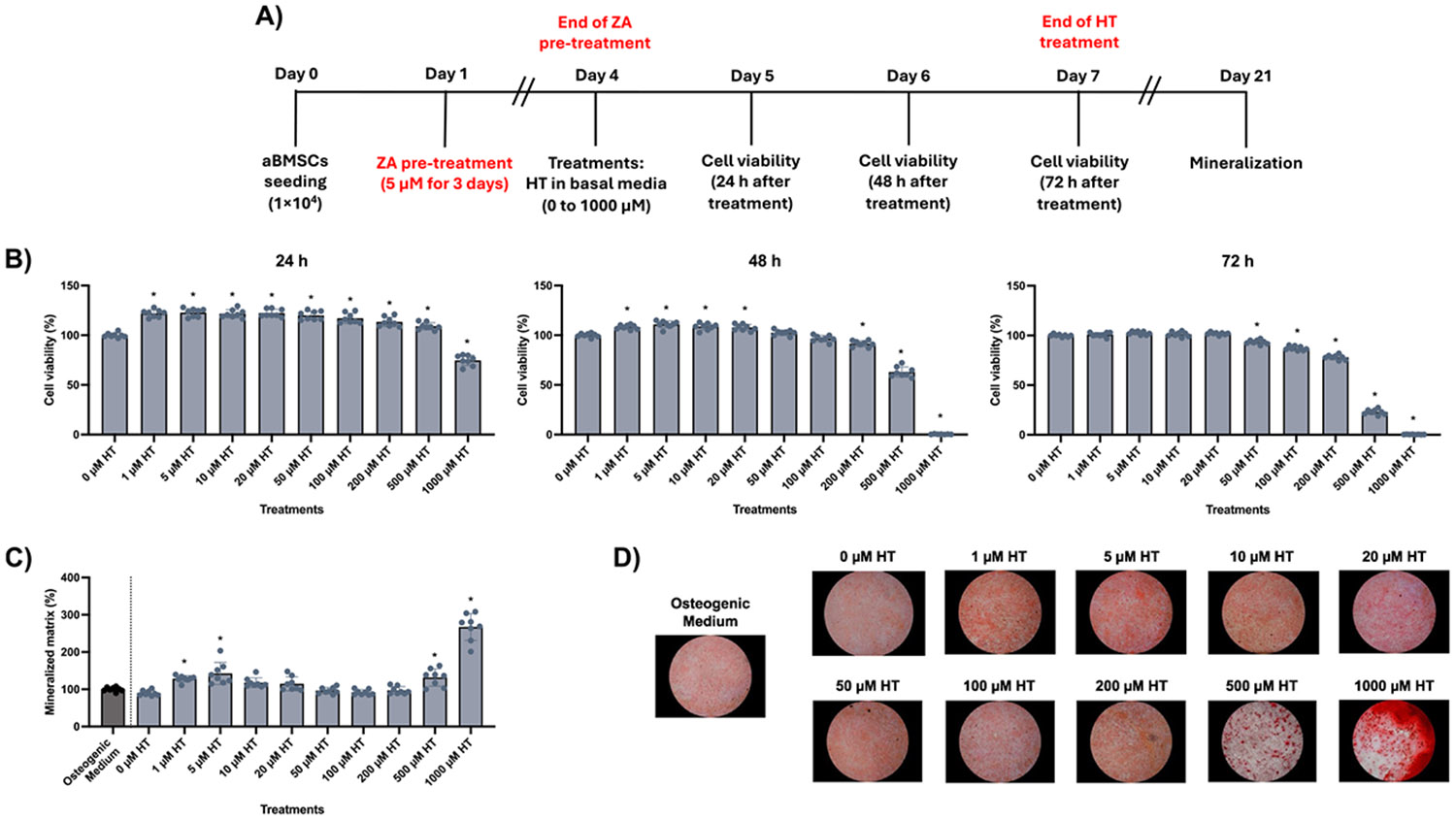
Modulatory effects of hesperetin (HT) on zoledronic acid (ZA)-challenged alveolar bone-derived mesenchymal stem cells (aBMSCs). **(A)** Experimental design: Cells were seeded and treated with 5 μM ZA for 3 days. Then, the medium was replaced with basal medium containing varying concentrations of HT for 72 h. Cell viability was assessed 24, 48, and 72 h after the HT exposure, which was renewed daily. After that, treatments were replaced with osteogenic medium (OM), which was renewed every other day, and mineralization was evaluated 21 days after cell seeding. **(B)** Cell viability and **(C)** mineralization results. Data are presented as means ± SD (n = 8) and calculated as a percentage of 0 μM HT for cell viability and osteogenic medium for mineralization, respectively, as controls. Asterisks indicate statistical difference compared to the respective controls (One-way ANOVA/Dunnett’s test, α=5 %). **(D)** Representative images of cells stained with Alizarin red solution, showing the red calcium deposits.

Subsequently, we investigated whether HT could modulate ZA-induced alterations in aBMSCs (Fig. 3A). HT exposure for 72 h was not cytotoxic at concentrations up to 200 μM (≥ 78.1% cell viability), while higher concentrations (≥ 500 μM) reduced metabolic activity by more than 70 % (Fig. 3B). Pre-treatment with 5 μM ZA for 3 days significantly affected mineralization even under osteogenic stimulation. HT treatment partially attenuated this effect, with 1–5 μM increasing mineralization levels compared to controls. Higher concentrations (≥ 500 μM) remained cytotoxic (Fig. 3C-D).

Taken together, these results demonstrate that HT promoted mineralized matrix deposition in healthy aBMSCs and partially restored osteogenic capacity under ZA challenge. Based on these findings, concentrations between 5 and 20 μM were considered relevant and selected for subsequent experiments addressing inflammatory conditions.

### 3.3. Establishment of an in vitro inflammatory microenvironment and modulation by hesperetin

**Fig. 4.**
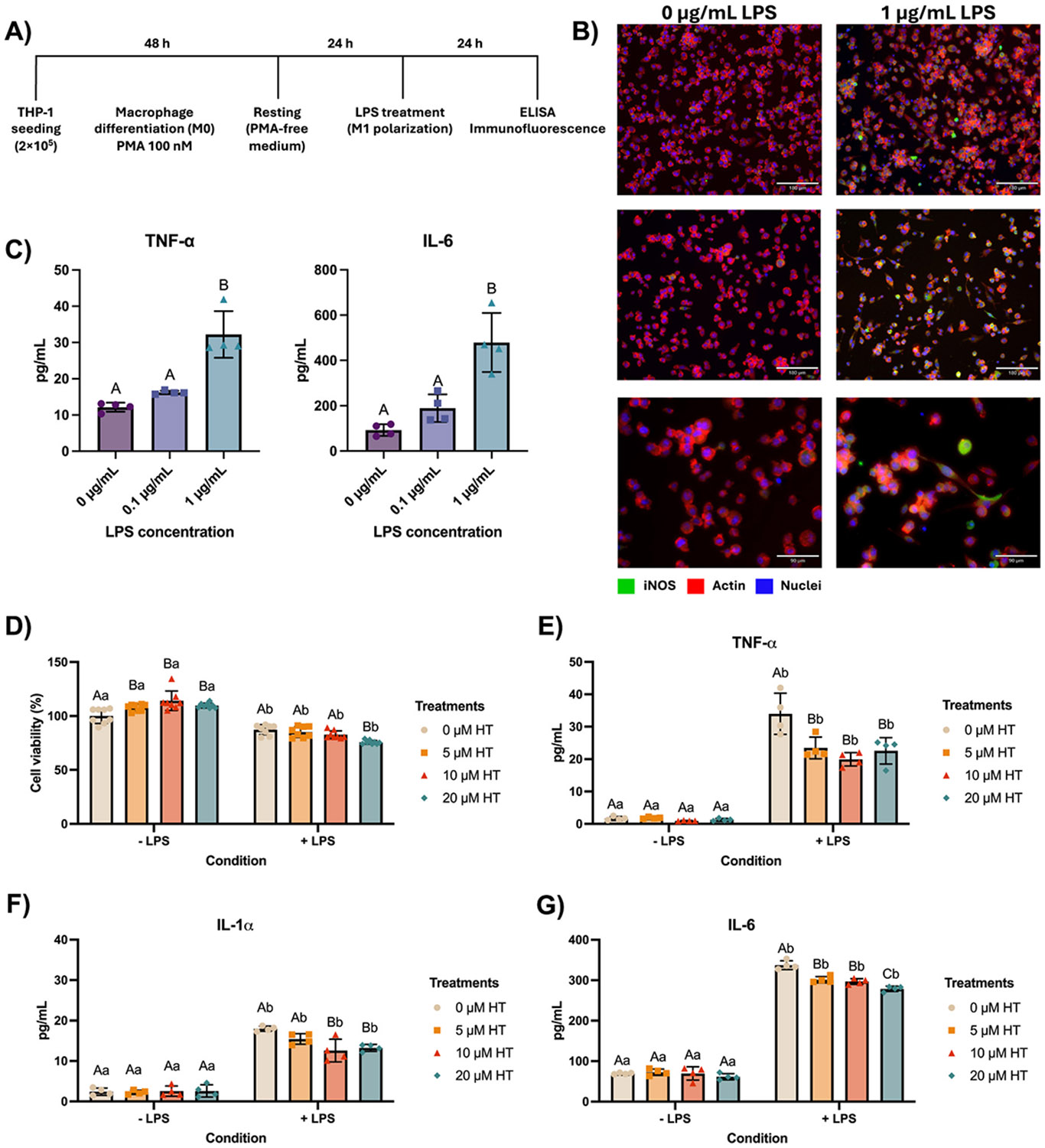
Establishment of an *in vitro* inflammatory microenvironment and modulation by hesperetin. **(A)** Experimental design for macrophage differentiation (M0 phenotype) and M1 polarization from the THP-1 monocytic cell line. **(B)** Immunofluorescence showing the expression of iNOS (M1 polarization marker) 24 h after LPS treatment. **(C)** Synthesis of TNF-α and IL-6 by M1-polarized THP-1-derived macrophages 24 h after LPS treatments. Data are presented as means ± SD (n = 4). Different letters denote statistically significant differences (One-way ANOVA with Tukey’s test, α=5 %). **(D)** Cell viability, and synthesis of **(E)** TNF-α, **(F)** IL-1α, and **(G)** IL-6 by M1-polarized THP-1-derived macrophages 24 h after hesperetin (HT) treatments combined (+LPS) or not (−LPS) with 1 μg/mL LPS. Data are presented as means ± SD (n = 4). For all graphs, uppercase letters indicate statistical comparisons between treatments within the conditions (−LPS or +LPS), while lowercase letters compare treatments across different conditions (−LPS or +LPS). Different letters denote statistically significant differences (Two-way ANOVA/Sidak’s test, α=5 %).

To establish the inflammatory model, THP-1-derived macrophages were polarized to the M1 phenotype by LPS stimulation. Immunofluorescence analysis confirmed M1 polarization by the expression of iNOS 24 h after LPS exposure (Fig. 4B). In parallel, LPS significantly increased the secretion of TNF-α and IL-6 compared to unstimulated controls, validating the pro-inflammatory condition with 1 μg/mL (Fig. 4C).

The modulatory effects of HT were assessed in M1-polarized macrophages. HT treatment did not impair cell viability in the absence of LPS, with the higher concentration (20 μM) reducing the metabolic activity by less than 25 %, thus being not considered cytotoxic (Fig. 4D). Under LPS stimulation, HT at all concentrations significantly reduced TNF-α and IL-6 secretion compared to LPS-only controls. However, only HT at 20 μM reduced IL-1α secretion (Fig. 4E-G). These results indicated that HT exerts a dose-dependent immunomodulatory effect by attenuating pro-inflammatory cytokine secretion in M1 macrophages. Based on these outcomes, 20 μM HT was selected for subsequent co-culture experiments to balance efficacy and cytocompatibility.

### 3.4. Modulatory effects of hesperetin in the inflammatory co-culture model

**Fig. 5.**
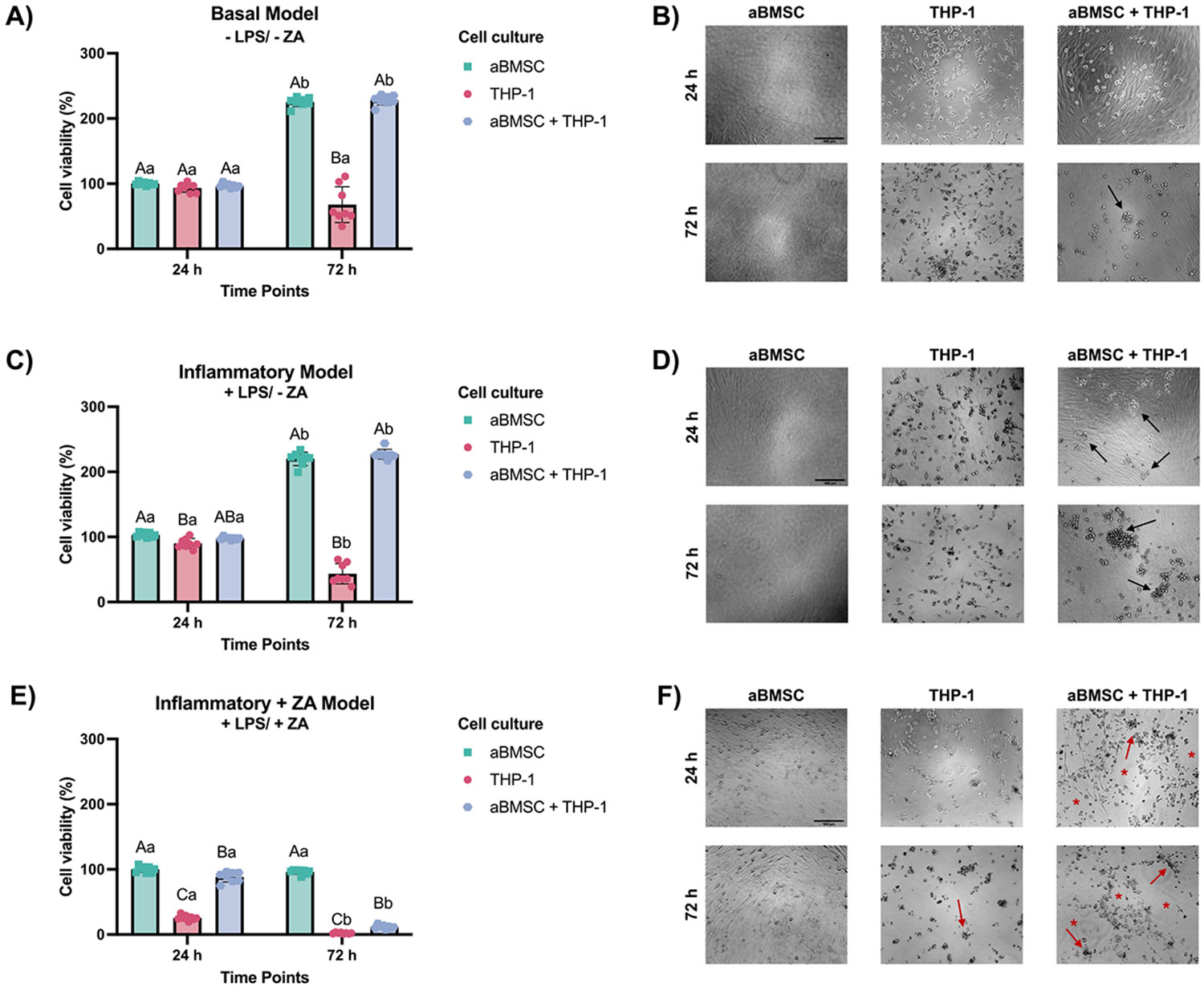
Establishment of a Co-Culture System to Mimic Inflammatory and ZA-Challenged Bone Microenvironments. **(A, B)** Cell viability and representative microscopy of aBMSCs and THP-1 cells under basal conditions, cultured either alone or in co-culture, at 24 h and 72 h. **(C, D)** Effects of LPS challenge (1 μg/mL) on cell viability and morphology at 24 h and 72 h. **(E, F)** Effects of ZA pre-treatment (5 μM, 3 days) in aBMSCs followed by LPS challenge on cell viability and morphology at 24 h and 72 h. Scale bar = 460 μm for all images. Black arrows indicate increased THP-1 cell rounding and clustering following inflammatory stimulation. Red arrows indicate pronounced morphological disruption, including regions with increased cellular debris. Red asterisks indicate areas of reduced aBMSC density and loss of confluence, particularly following the combined ZA and LPS challenge. Data are presented as mean ± SD (n = 8). Uppercase letters indicate comparisons between culture conditions at the same time point, while lowercase letters indicate comparisons across time points. Different letters denote statistically significant differences (Two-way ANOVA/Sidak’s test, α = 0.05).

To further explore the osteoimmunomodulatory effects of HT, a coculture model combining aBMSCs and THP-1-derived macrophages was established. Under basal conditions, both cell types preserved high viability and their characteristic morphology when cultured alone or in co-culture, with aBMSCs displaying a well-spread, elongated fibroblastlike morphology and THP-1-derived macrophages exhibiting a rounded to polygonal adherent morphology with short cytoplasmic processes, consistent with an M0 phenotype (Fig. 5A-B). LPS challenge significantly reduced cell viability and induced time-dependent morphological alterations at both 24 h and 72 h compared with unstimulated controls, including reduced aBMSC confluence, increased cellular rounding, loss of cytoplasmic extensions in macrophages, and enhanced cell aggregation, particularly evident at 72 h (Fig. 5C-D). When ZA-pretreated aBMSCs were included in the co-culture followed by LPS stimulation, a marked cytotoxic effect was observed, characterized by increased cellular debris, reduced cell density, and pronounced disruption of aBMSC confluence, especially under co-culture conditions, thereby precluding further analyses under this condition (Fig. 5E-F).

**Fig. 6.**
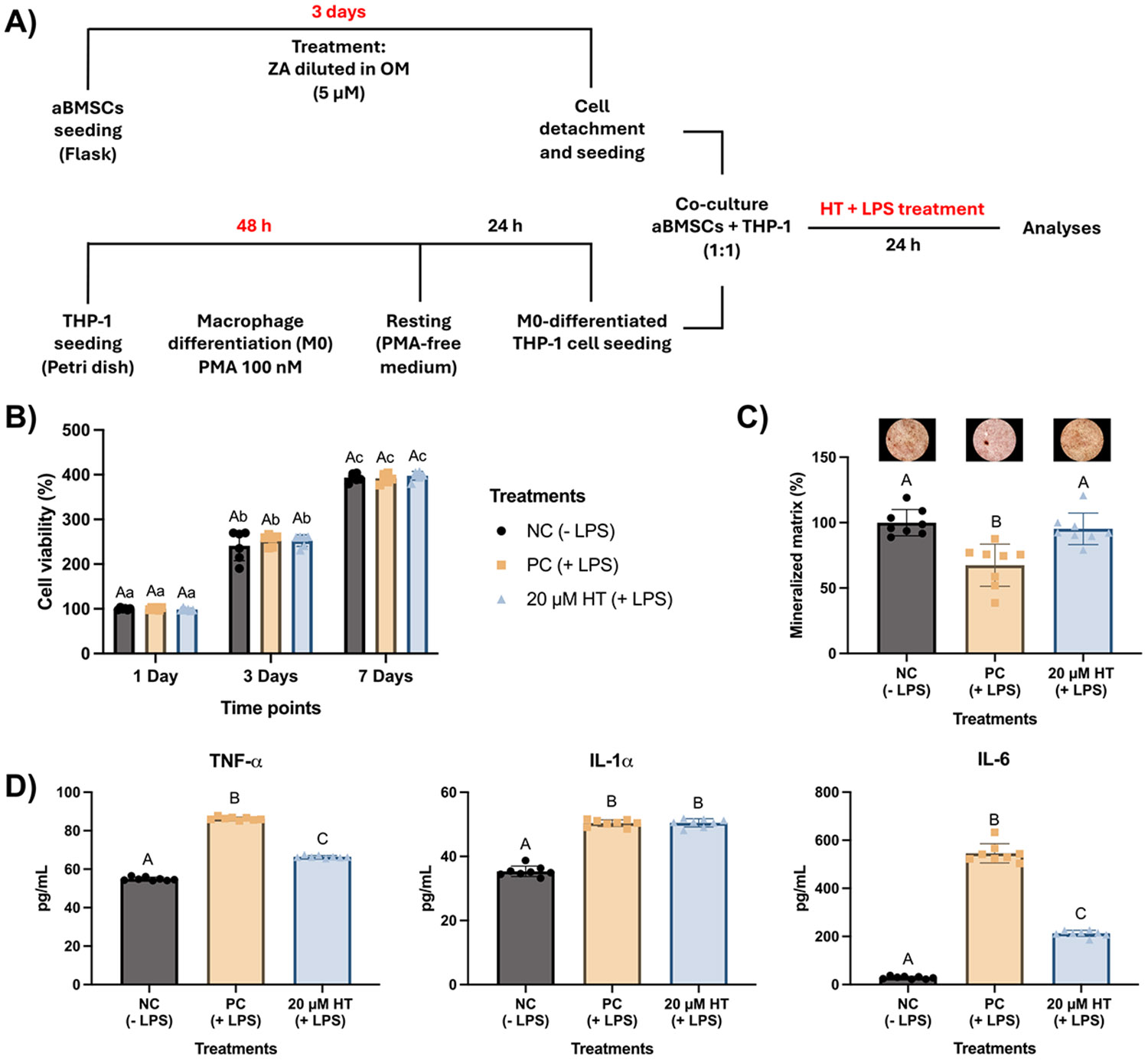
Modulatory effects of hesperetin on the inflammatory co-culture system **(A)** Experimental design: aBMSCs and THP-1 were grown individually for 3 days. The aBMSCs were cultured in osteogenic medium (OM medium), while THP-1 cells were differentiated into macrophages (M0 phenotype). Then, both cells were cocultured and either stimulated with LPS for an additional 24 h (PC) or not (NC). Cells stimulated with LPS were simultaneously treated with hesperetin (20 μM HT). Cells were exposed to the treatments for 24 h only and cultured for up to 21 days. **(B)** Cell viability after 1, 3, and 7 days. Data are presented as means ± SD (n = 8). Uppercase letters indicate statistical comparisons between treatments within the same time point, while lowercase letters compare treatments across different time points. Different letters denote statistically significant differences (Two-way ANOVA/Sidak’s test, α=5 %). **(C)** Mineralized matrix formation after 21 days, and **(D)** synthesis of TNF-α, IL-1α, and IL-6 by 24 h after LPS treatments. Data are presented as means ± SD (n = 8). Different letters denote statistically significant differences (One-way ANOVA/Tukey’s test, α=5 %).

Given the high cytotoxicity of the inflammatory + ZA model, only the LPS-induced inflammatory co-culture was carried forward to assess HT modulation (Fig. 6A). HT at 20 μM was selected based on previous monoculture results. In this setting, HT did not impair cell viability over 7 days (Fig. 6B). After 21 days, mineralized matrix deposition was significantly higher in the HT-treated group compared with the LPS-only control (Fig. 6C). In parallel, HT treatment significantly reduced the secretion of TNF-α and IL-6 after 24 h compared to LPS-stimulated cocultures without HT, whereas IL-1α was not significantly affected under these conditions (Fig. 6D). These findings demonstrate that HT was able to attenuate the inflammatory response in macrophages while supporting mineralized matrix formation by aBMSCs within the co-culture system.

## 4. Discussion

This study provides initial evidence that HT administration may be a valuable adjuvant therapy to counteract bisphosphonate-associated MRONJ and enhance bone healing by integrating mineralization and immunomodulatory effects. In healthy aBMSCs, HT at intermediate concentrations promoted mineralized matrix deposition without impairing cell viability. Under the ZA challenge, HT partially restored mineralization, indicating a protective effect against bisphosphonate-induced dysfunction. Additionally, HT reduced pro-inflammatory cytokine synthesis in M1 macrophages and preserved mineralization in the inflammatory co-culture model. Together, these findings demonstrate that HT can modulate both osteogenic and immune responses, two critical processes for maintaining bone homeostasis under adverse conditions.

Two experimental strategies were employed to establish ZA preconditioning in aBMSCs. Prolonged exposure (1–50 μM for 7 days) induced pronounced cytotoxicity and almost completely abolished mineralization, in line with previous reports of ZA’s potent inhibitory effects on osteogenic cells. Concentration- and time-dependent decreases in cell viability, mineralized nodule formation, ALP activity, and expression of osteogenic genes (Runx2, Col I, ALP, OCN) have been demonstrated in both bone progenitor cells and human osteoblasts (Basso et al., 2013; Patntirapong et al., 2012). More recent evidence indicates that ZA can differentially influence osteogenesis, affecting early markers such as ALP, BMP4, and OCN, while still markedly inhibiting mineralized nodule formation (Wang et al., 2024).

Importantly, although ZA at 10 and 50 μM markedly reduced cell viability at days 7 and 14, this did not prevent the detection of mineral deposits at later time points. As the Alizarin Red stains calcium deposits retained on the plate and does not require ongoing metabolic activity at the time of analysis, the mineralization observed at days 14 and 21 likely reflects early deposits formed before the collapse in viability, which remain attached to the matrix and are preserved through fixation. This interpretation is consistent with the pronounced cytotoxicity detected in the intermediate periods. This suggests that the mineralization phase is sensitive to ZA, highlighting the importance of assessing final functional outcomes (mineralized matrix deposition) rather than relying solely on early molecular markers. Based on these findings, a refined protocol using shorter exposure (0.5–10 μM for 3 days) was set, demonstrating preserved cell viability while still impairing mineralization, thus providing a physiologically relevant model. Notably, the 5 μM concentration chosen for subsequent experiments corresponds to levels detected in the saliva of patients receiving chronic ZA therapy (Scheper et al., 2009), further supporting the translational relevance of our system. This strategy enabled the evaluation of HT’s capacity to mitigate ZA-induced osteogenic impairments while preserving overall cellular function.

Before testing HT under ZA challenge, its effects were first characterized in healthy aBMSCs following a short-term, 3-day exposure. Osteogenic differentiation of aBMSCs was assessed functionally by matrix mineralization analysis. An osteogenic medium was included as a positive control and induced a marked increase in mineralized matrix deposition compared with basal medium, confirming the osteogenic differentiation capacity of aBMSCs. HT promoted mineralization at intermediate concentrations (specifically 20 μM) without impairing viability, reinforcing its intrinsic pro-osteogenic potential. These results align with earlier studies showing that HT stimulates osteogenic differentiation through canonical signaling pathways, including ERK, Smad1/5/8, PI3K/AKT/mTOR, and Wnt/β-catenin (Kim et al., 2013; Liu et al., 2021; Xie et al., 2023; Xue et al., 2017). Rather than replicating these well-documented mechanisms, our study focused on a more integrated and biologically relevant endpoint: mineralized matrix deposition. By emphasizing terminal cellular responses rather than intermediate signaling events, we provide direct evidence of HT’s ability to sustain osteogenesis under different conditions.

Importantly, HT was applied as an early modulatory stimulus limited to a 3-day exposure, rather than as a continuous differentiation factor, because matrix mineralization represents a cumulative downstream outcome that does not necessarily require sustained compound presence once early osteogenic signaling has been triggered (Mendes Soares et al., 2024). Accordingly, HT was intentionally discontinued after the initial exposure period to assess whether its early effects were sufficient to support long-term mineral deposition, reducing possible cytotoxic effects due to long-term exposure. This approach strengthens translational relevance, highlighting its potential for clinically feasible, brief treatment regimens, including local application during surgical procedures, short-term adjuvant therapy, or incorporation into biomaterials with controlled release properties to provide sustained delivery at the target site and support bone repair without requiring prolonged direct exposure *in vitro*.

The protective effect of HT became particularly relevant under ZA challenge. As expected, ZA exposure significantly impaired mineralization in aBMSCs, confirming its deleterious impact on progenitor cell function. It is also important to note that high HT concentrations could exert a strong pro-mineralizing influence while remaining considerably toxic, a dual effect that mirrors what we observed for ZA, where early mineral deposition occurred despite subsequent loss of viability. As previously discussed, mineral deposits formed before metabolic collapse may persist and be detected despite reduced viability, reflecting the cumulative nature of Alizarin Red staining rather than ongoing osteogenic activity. Conversely, intermediate HT concentrations partially restored mineralization, indicating that it can mitigate, but not fully overcome, bisphosphonate-induced dysfunction. Clinically, these findings are relevant given the increased risk of poor bone healing, MRONJ triggering, delayed osseointegration, and implant failure among patients receiving ZA therapy (H. Liu et al., 2025; Paschalidi et al., 2021; Roato et al., 2023; Sulaiman et al., 2023). However, the partial recovery underscores the multifactorial nature of ZA effects, which involve not only osteogenic suppression but also vascular and immune dysregulation that cannot be addressed solely at the level of progenitor cell differentiation (Jelin-Uhlig et al., 2024).

The immunomodulatory effects of HT were equally notable. In M1-polarized macrophages, HT significantly reduced the synthesis of TNF-α, IL-6, and IL-1α, demonstrating its anti-inflammatory action in a dosedependent manner. These results align with prior evidence that HT suppresses the NF-κB and AMPK/p53 signaling pathways (Mendes Soares et al., 2024; Xie et al., 2023), while other flavonoids exert similar effects on the innate immune response (Jomova et al., 2025). Given that chronic activation of M1 macrophages is a major contributor to impaired bone regeneration and MRONJ pathogenesis (Paschalidi et al., 2021; Roato et al., 2023), the ability of HT to attenuate cytokine release directly addresses a key pathogenic mechanism. As the underlying molecular pathways of flavonoids on inflammation are well documented (Jomova et al., 2025), our study prioritized functional outcomes. By assessing cytokine synthesis, we directly evaluated the net impact of HT on modulating the proinflammatory environment. This emphasis is particularly relevant in the framework of osteoimmunology, where the quality and magnitude of cytokine release determine whether immune activation supports or hinders tissue regeneration (Mori et al., 2013). Nevertheless, the use of THP-1-derived macrophages limits the extrapolation of our findings, as this model does not fully reproduce the heterogeneity of myeloid and lymphoid subsets that orchestrate immune responses in vivo.

The co-culture model offered an intermediate level of complexity by integrating osteoprogenitor cells and macrophages in the same environment (Ren et al., 2023; Song et al., 2020). This model is useful because THP-1 cells capture key macrophage responses, and aBMSCs represent human osteoprogenitors, allowing us to test how HT affects both bone and immune cells, reflecting the central role of monocytes and macrophages as critical mediators of stromal cell-driven immunomodulation (Ren et al., 2023). Under inflammatory stimulation with LPS, co-cultures showed reduced mineralization and increased cytokine release, recapitulating the detrimental effects of chronic inflammation on bone repair. Consistent with these functional outcomes, LPS exposure induced visible morphological stress in co-cultures, characterized by reduced stromal confluence and increased macrophage clustering. Importantly, HT preserved mineralization and suppressed cytokine secretion in this inflammatory setting, supporting its dual osteogenic and anti-inflammatory actions within a more physiologically relevant system. These findings have direct translational implications for clinical conditions characterized by inflammation-driven bone loss, including periodontitis, peri-implantitis, and MRONJ. In contrast, the combined ZA and LPS challenge resulted in severe structural disruption and cellular debris accumulation, reflecting the excessive aggressiveness of this condition for monolayer *in vitro* modeling and limiting the maintenance of viable co-cultures. While this limitation prevents conclusions about HT efficacy in the most severe clinical scenarios, it also highlights the need for more robust three-dimensional models capable of mimicking the synergistic stress imposed by pharmacological and inflammatory insults.

Overall, the present study positions HT as an osteoimmunomodulatory molecule capable of sustaining *in vitro* cell-related bone regeneration events under adverse conditions. By focusing on functional outcomes (mineralized matrix deposition and cytokine secretion), our approach integrates well-established mechanistic knowledge with cellular endpoints relevant to clinical repair dynamics. From a translational perspective, these data support the rationale for exploring HT as an adjuvant strategy to enhance bone regeneration or mitigate inflammation-mediated bone loss. Preclinical evidence shows that HT exerts antioxidant, anti-inflammatory, cardioprotective, neuroprotective, and osteoprotective actions across multiple experimental models, indicating a broad therapeutic potential (Salehi et al., 2022). This perspective emphasizes the translational potential of HT as a candidate adjuvant therapy for bone regeneration and MRONJ prevention, particularly because oxidative stress and inflammation have been implicated in MRONJ pathogenesis *in vivo* and in humans (Bagan et al., 2013, 2014; de Barros Silva et al., 2016, 2017), and antioxidant agents have shown protective effects in relevant models (Cavalcante & Tomasetti, 2020; Vitale et al., 2024). However, its current clinical evidence is restricted and does not include oral applications, nor is it commercially available in formulations suitable for clinical use. As our study did not evaluate oxidative-stress pathways, the relevance of HT’s antioxidant properties to MRONJ remains to be confirmed.

Important limitations should be acknowledged. The study was conducted entirely *in vitro*, relied on simplified 2D and co-culture systems, and did not include biomaterial-based delivery platforms or pharmacologically relevant dosing regimens. Although HT is naturally occurring and has been evaluated for safety in nutritional or other contexts, its limited systemic bioavailability is highlighted as a major barrier to clinical translation. Furthermore, *in vivo* experimentation is needed to confirm HT’s therapeutic potential, particularly under ZA exposure and chronic inflammation. Future studies should therefore focus on developing effective delivery systems for HT, testing its efficacy in animal models of compromised bone repair, and exploring its integration with established regenerative strategies. These steps will be essential to determine whether the dual osteogenic and immunomodulatory effects observed in vitro can translate into clinically meaningful outcomes.

## 5. Conclusion

Collectively, our findings highlight hesperetin as an osteoimmuno-modulatory molecule capable of sustaining *in vitro* cell-related bone regeneration responses under zoledronic acid and inflammatory stress. The present data support its translational potential as a promising adjuvant strategy against bisphosphonate-associated MRONJ and for enhancing bone healing outcomes.
